# Clinical and Molecular Clues to Diagnosing Hereditary Hyperferritinemia-Cataract Syndrome: Case Report and Literature Review

**DOI:** 10.3390/genes16111381

**Published:** 2025-11-13

**Authors:** Barbora Ludikova, Lucie Sochorcova, Damjan Jaksic, Katarina Hlusickova Kapralova, Monika Horvathova

**Affiliations:** 1 Department of Pediatrics, Faculty of Medicine and Dentistry, Palacky University and University Hospital Olomouc, 779 00 Olomouc, Czech Republic; 2 Department of Biology, Faculty of Medicine and Dentistry, Palacky University, 779 00 Olomouc, Czech Republic; 3 Department of Pediatrics, T. Bata Regional Hospital, 762 75 Zlin, Czech Republic

**Keywords:** hyperferritinemia, cataract, *FTL* gene, IRP, HFE

## Abstract

**Background:** Hereditary hyperferritinemia-cataract syndrome (HHCS) is a rare autosomal dominant disorder characterized by persistently elevated serum ferritin and early-onset bilateral cataracts in the absence of systemic iron overload. It is caused by pathogenic variants in the iron-responsive element (IRE) of the *FTL* gene, leading to dysregulated L-ferritin synthesis. **Methods:** We evaluated a 12-year-old Czech girl with markedly elevated serum ferritin identified incidentally during workup for abdominal pain. Clinical assessment included biochemical, radiological, ophthalmological, and genetic testing of the proband and available family members. **Results:** Magnetic resonance imaging excluded systemic iron overload, while ophthalmological evaluation revealed bilateral cataracts. Family history indicated multiple affected relatives across three generations. Genetic testing confirmed a heterozygous *FTL* c.-168G>C variant. Additional screening for common *HFE* variants revealed heterozygous H63D in several family members, with no impact on ferritin or hepcidin levels. Beyond this case, we provide a comprehensive review of HHCS, including molecular mechanisms, an updated overview of reported *FTL* mutations, and ophthalmological features that distinguish HHCS cataracts from other congenital cataracts. **Conclusions:** This report underscores the translational relevance of combining molecular diagnostics, clinical evaluation, and family screening to improve recognition and management of HHCS, and to prevent misdiagnosis and unnecessary iron-depletion therapy.

## 1. Introduction

Hereditary hyperferritinemia-cataract syndrome (HHCS, OMIM #600886) is a rare autosomal dominant disorder characterized by persistently elevated serum ferritin and early-onset bilateral cataracts without systemic iron overload. The condition is caused by pathogenic variants in the iron-responsive element (IRE) within the 5′ untranslated region (UTR) of the *ferritin light chain* (*FTL*) gene located on chromosome 19, which encodes the light (L)-ferritin subunit [1,2,3]. Under physiological conditions, the IRE forms a stem-loop structure that binds iron regulatory proteins (IRPs), thereby inhibiting translation of *FTL* mRNA when cellular iron levels are low [4]. In HHCS, pathogenic variants disrupt this regulatory mechanism, leading to constitutive overproduction of L-ferritin.

Clinically, aside from isolated hyperferritinemia, the hallmark manifestation is progressive, bilateral cataract formation, usually developing in childhood or adolescence and often requiring surgical intervention [5]. This results from excessive accumulation of L-ferritin in the lens, with characteristic crystalline deposits that scatter light and impair vision. The severity of cataracts and serum ferritin levels may correlate with the specific IRE mutation [2]. The estimated prevalence of HHCS is around 1 in 200,000 individuals, though this is likely underestimated due to underdiagnosis, especially in milder or subclinical cases [1]. Fewer than 200 families have been reported worldwide, often identified incidentally during evaluation of unexplained hyperferritinemia [6,7]. Diagnosis relies on the detection of persistently elevated serum ferritin with otherwise normal iron parameters (serum iron, transferrin saturation, and total iron-binding capacity), absence of inflammation or liver disease, and the presence of early-onset bilateral cataracts. Definitive diagnosis is established by identifying a pathogenic variant in the *FTL* IRE region through molecular testing [1,2,3].

Our study highlights the diagnostic and clinical relevance of HHCS by integrating molecular, biochemical, and ophthalmologic data in a well-characterized Czech family and situates these findings within the broader context of reported *FTL* IRE variants. Although HHCS is a well-described condition, it is still frequently misdiagnosed, which may lead to inappropriate iron-reducing therapy and delays in genetic counseling, underscoring the need for continued clinician education.

## 2. Materials and Methods

### 2.1. Clinical and Biochemical Analyses

Clinical history and laboratory data, including ophthalmological examinations and molecular biology studies, were collected. Hematological parameters were measured using a Sysmex XE-500 analyzer (Sysmex Corp, Kobe, Japan). Iron-related parameters were assessed as follows: serum iron (sFe) and total iron-binding capacity (TIBC) were determined by colorimetric assays on an automated clinical chemistry analyzer (F. Hoffmann-La Roche Ltd., Basel, Switzerland), while serum ferritin was measured by chemiluminescent immunoassay using the Immulite 2000 XPi analyzer (Siemens Healthcare Diagnostics, Malvern, PA, USA). Transferrin saturation was calculated as the ratio of sFe to TIBC, expressed as a percentage. Hepcidin serum concentrations were measured with an ELISA kit (DRG Instruments GmbH, Marburg, Germany), as we previously described [8]. The proband also underwent magnetic resonance imaging (MRI) of the liver and other parenchymal organs.

### 2.2. Targeted Sanger Sequencing

Blood samples were obtained from the patient and family members after informed consent. Genomic DNA was extracted using the Gentra Puregene Blood kit (Qiagen, Venlo, The Netherlands). The target sequence of the *FTL* gene (NM_000146.4; 5′UTR and exon 1) was amplified using the HotStartTaq Master Mix kit (Qiagen) and specific *FTL* primers: forward primer CACCATAAAAGAAGCCGCCC, reverse primer AGCTGGAGGAAATTAGGGCCA. The exons 2 and 4 of the *HFE* gene (NM_000410.3) were amplified using the following set of primers: forward primer GGCCTGTTGCTCTGTCTCCA, reverse primer AAAGCTCTGACAACCTCAGGAAGG for exon 2 and forward primer AAAGGGTATTTCCTTCCTCCAACC, reverse primer GCAGATCCTCATCTCACTGCCA for exon 4. PCR reactions were performed using the MJ Mini Thermal Cycler (Bio-Rad, Hercules, CA, USA). After PCR, enzymatic purification was performed using exonuclease I and alkaline phosphatase (both from ThermoFisher, Waltham, MA, USA) according to the manufacturer’s protocol. The purified PCR products were subjected to conventional Sanger sequencing (StandardSeq, SEQme company, Dobris, Czech Republic) using the same forward and reverse primers as for amplification. Sanger sequencing chromatograms were analyzed using FinchTV 1.4.0 software (Geospiza, Inc., Seattle, WA, USA).

### 2.3. Literature Review Methodology

The accompanying literature review was conducted as a narrative synthesis of published HHCS cases identified through PubMed and Scopus searches (no date restrictions). Publications describing *FTL* IRE variants, clinical presentation, and ophthalmological findings were included and reviewed to provide an updated overview of molecular and phenotypic characteristics.

## 3. Results

### 3.1. Clinical Findings and Biochemical Analyses

A 12-year-old girl was evaluated for nonspecific abdominal pain, during which incidental laboratory testing revealed markedly elevated serum ferritin levels (1418 ± 265 ng/mL). Other iron-related parameters, including serum iron, transferrin saturation, and total iron-binding capacity, were within the normal range (Table 1). There was no clinical or biochemical evidence of inflammation, and MRI showed no pathological iron deposition.

**Figure 1:**
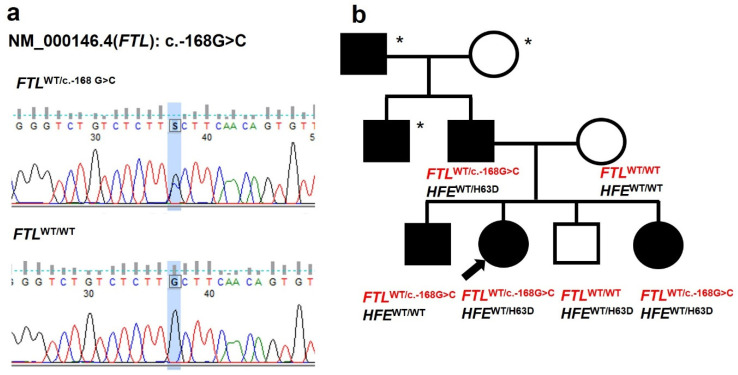
Genetic and pedigree analysis. (**a**) The sequencing chromatogram shows the c.-168G>C mutation (marked by a blue rectangle) in the 5′ untranslated region (IRE) of the *FTL* gene found in the proband. (**b**) A three-generation family pedigree with HHCS. Black symbols represent individuals with HHCS. Open symbols indicate unaffected individuals. Asterisks mark subjects who have not undergone genetic testing. The arrow points to the proband. *HFE* genotype information is provided for the tested family members.

Detailed family history revealed hyperferritinemia and cataracts in multiple relatives across three generations on the paternal side (Figure 1). Ophthalmic examination of the proband documented a bilateral decrease in best-corrected visual acuity, numerous small lens opacities, and marked hyperopia. Biochemical analyses of available family members with cataracts (father and two siblings) confirmed isolated elevation of ferritin (Table 1). Importantly, ophthalmological history confirmed that cataracts had been present since the neonatal period in the affected children, while the father’s cataracts manifested in early childhood. Surgical intervention was required in both the proband and her father; her affected brother is currently awaiting surgery. Despite this consistent familial pattern, HHCS was neither suspected nor diagnosed until genetic testing for unexplained hyperferritinemia in the proband was performed.

### 3.2. Genetic Testing

**Figure 2:**
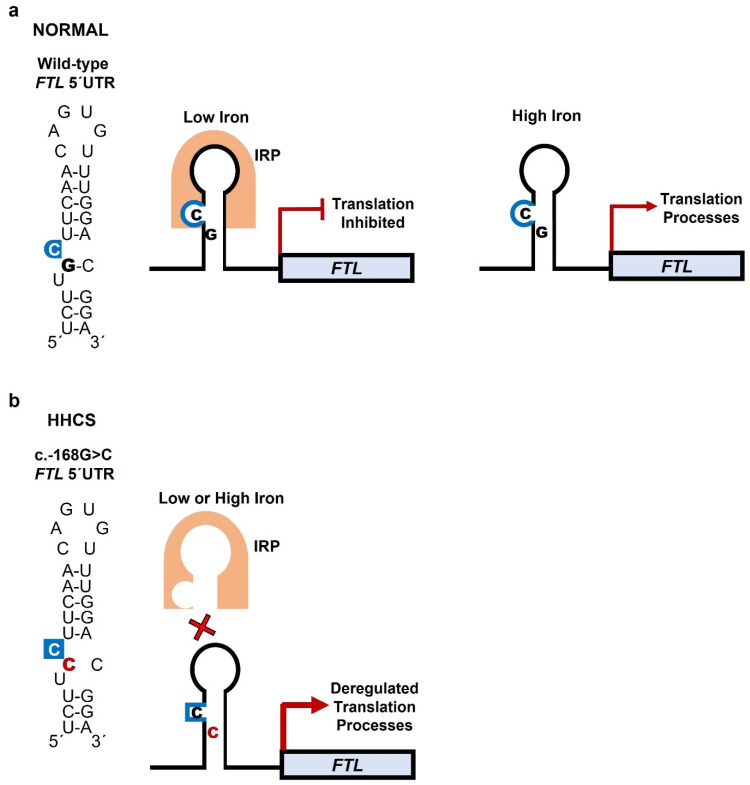
Mechanism of IRP binding to *FTL* mRNA under normal and mutant conditions. The IRE, located in the 5′ UTR of *FTL* mRNA, forms a stem–loop (hairpin) RNA structure. The stem consists of complementary base pairs but contains several mismatches; among these, the highly conserved cytosine (C) bulge is essential for high-affinity IRP binding. This bulge divides the stem into upper and lower segments and, together with the loop hexanucleotide sequence (CAGUGN), provides the key recognition motifs for IRP binding. (**a**) Wild-type IRE: Under iron-deficient conditions, IRPs bind to the IRE, blocking ribosome recruitment and suppressing *FTL* translation to maintain iron availability. When iron is high, IRPs do not bind to the IRE, translation proceeds, and ferritin is produced to store excess iron. (**b**) Mutant IRE: The c.-168G>C variant alters base pairing in the stem, disrupting C-bulge geometry and hairpin conformation. This reduces IRP binding, leading to dysregulated, iron-independent overproduction of ferritin.

Targeted Sanger sequencing of the patient and family members identified a heterozygous pathogenic variant in the *FTL* gene: (*FTL*): c.-168G>C (rs398124635) in the proband, her father, older brother, and younger sister (Figure 1, Table 1). In the wild-type sequence, the G nucleotide at position -168 pairs within the stem to stabilize the IRE hairpin and maintain the bulged cytosine (C) at position -167, which is crucial for IRP binding. The G to C substitution disrupts base pairing and likely alters the C bulge geometry, impairing IRP binding [9] (Figure 2, Table 2). This mutation has also been previously reported in an unrelated Czech family [10]. To date, two other *FTL* mutations have been described in Czech families: a de novo c.-167C>T and an inherited c.-161C>T [10]. Considering the estimated prevalence of HHCS (~1 in 200,000), additional cases are expected in the Czech Republic; however, many patients are likely undiagnosed or misdiagnosed.

### 3.3. HFE Genotyping and Hepcidin Measurement

Due to persistently elevated ferritin levels, the proband’s father was misdiagnosed with hereditary hemochromatosis (HH) for many years [6,12]. Although genetic testing revealed the causative *FTL* mutation confirming HHCS, all available family members were also screened for the most common HH-associated mutations in *HFE*. Such testing, particularly for p.Cys282Tyr (C282Y) and p.His63Asp (H63D) variants, is advisable in patients of European origin, given the high frequency of these alleles in this population [12,13]. Three out of four affected family members carried the H63D variant in a heterozygous state (Figure 1, Table 1). The *HFE* H63D allele is a low-penetrance variant associated with HH mainly in homozygotes or compound heterozygotes with C282Y [6,12]. We observed no association between *HFE* genotype and ferritin levels in this family.

To further assess the potential impact of *HFE* variants on iron metabolism, we measured circulating hepcidin, the key hormone regulating systemic iron homeostasis [14]. Patients with HHCS generally have normal hepcidin levels, whereas in *HFE*-related HH, deregulated suppression of hepcidin drives iron overload [14]. In our family, hepcidin levels in affected members carrying the H63D variant did not differ from those of age-matched healthy controls (Table 1). Interestingly, the affected proband’s brother with the wild-type *HFE* showed the lowest hepcidin, suggesting a possible contribution of additional genetic or regulatory factors modulating hepcidin production [6,12,14].

## 4. Discussion

Hereditary hyperferritinemia cataract syndrome (HHCS) is a rare genetic disorder that poses diagnostic and management challenges. Its pathogenesis arises from the disruption of post-transcriptional regulation of *FTL* mRNA.

Ferritin, an intracellular iron storage protein, consists of light (L) and heavy (H) subunits. The L subunit predominates in storage tissues and plays a key role in long-term iron sequestration. Under iron-deficient conditions, ferritin synthesis is inhibited by IRPs binding to the IRE in the 5′ UTR of *FTL* mRNA [4]. Rising iron levels disrupt IRP–IRE binding, thereby permitting ferritin synthesis to match cellular needs (Figure 2). IREs contain a conserved hexanucleotide loop (5′-CAGUGN-3′) and a C bulge within the stem; both motifs are critical for IRP recognition and regulation of *FTL* translation. Mutations in the IRE alter its secondary structure, impair IRP binding, and result in constitutive overproduction of L-ferritin irrespective of iron status [3,4,15]. Excess L-ferritin accumulates particularly in the lens, forming crystalline aggregates that scatter light and impair transparency.

**Figure 3:**
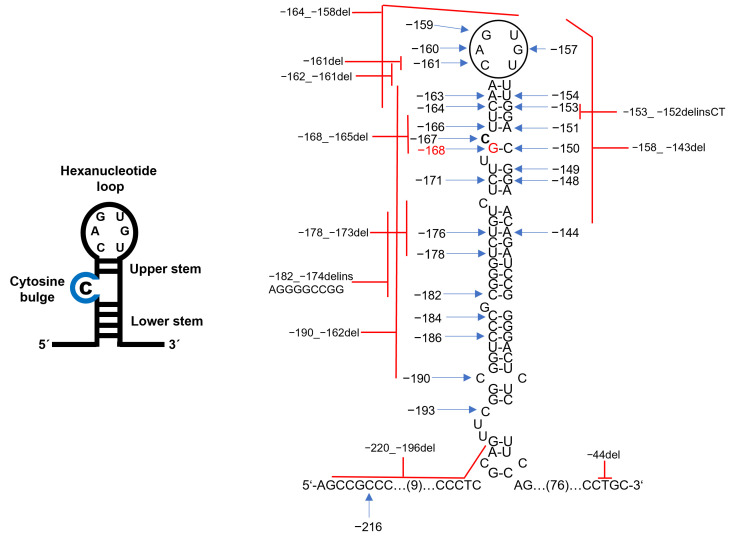
Pathogenic variants in the *FTL* IRE associated with HHCS. Schematic of the *FTL* mRNA IRE hairpin, showing the conserved hexanucleotide loop and C-bulge (blue) critical for IRP binding. The pathogenic point mutations (blue arrows) and deletions (red lines) are distributed across the loop, C-bulge, upper and lower stem, and stem base. The position of the c.-168G>C mutation identified in the studied family is indicated in red. The pathogenic variants variably alter IRE secondary structure and reduce IRP binding affinity. For details on individual variants, see Table 2.

To date, at least 47 different *FTL* mutations have been described, including single-nucleotide substitutions and small indels [15]. Table 2 summarizes all *FTL* IRE mutations described to date, cross-references their current HGVS nomenclature with previous designations, and outlines the proposed molecular mechanisms underlying their pathogenicity [11]. The most common is c.-160A>G (+40A>G) [15,16] (Figure 3, Table 2). Most HHCS-causing mutations are heterozygous, consistent with autosomal dominant inheritance. Homozygous mutations are rare but have been reported [2,17,18]. For c.-150C>A (+51G>C), homozygotes had ~2-fold higher ferritin than heterozygotes but only mild cataracts manifesting in adulthood [17]. Thus, phenotypic differences compared with heterozygotes were minimal. A positive family history of hyperferritinemia and early cataracts is common, though de novo cases are also reported [19,20].

In the Czech Republic, three *FTL* IRE variants have previously been reported: the de novo c.-167C>T and the inherited c.-161C>T and c.-168G>C [10] (Table 3). Together with our current family, these account for four independent HHCS pedigrees. The two families carrying the c.-168G>C variant originate from different regions of the country and have no known shared family ancestry. Moreover, the same *FTL* c.-168G>C variant has been reported in families outside the Czech Republic [2,21], supporting independent mutational events rather than a founder origin. Definitive evaluation of a potential founder effect would require detailed haplotype or microsatellite analysis to assess relatedness, which is beyond the scope of this study. In addition, alternative substitutions at the same nucleotide (c.-168G>A, c.-168G>T) [2,3] further indicate that this site represents a mutational hotspot within the IRE.

Although previously reported Czech HHCS patients exhibited elevated serum ferritin without iron overload and characteristic lens opacities [10], these cases also illustrate notable clinical variability and frequent diagnostic delays. Several probands were identified only after treatment for unrelated or misinterpreted conditions, including chelation therapy followed by seizure due to hyperammonemia, incidental detection during management of pulmonary embolism, or hyperferritinemia discovered during evaluation of joint pain and recurrent febrile episodes (Table 3) [10]. These examples highlight the risk of misdiagnosis and inappropriate management before genetic confirmation. In contrast, none of our affected family members exhibited systemic complications; only the proband reported transient abdominal pain, with no evidence of iron overload or other organ involvement.

Phenotypic variability in HHCS is well recognized. Some individuals remain asymptomatic aside from subtle lens opacities, whereas others develop visual impairment requiring early cataract surgery. HHCS cataracts are typically bilateral with numerous white punctate opacities scattered in the cortex (Table 4) [21,22]. These crystalline patterns are highly specific and help distinguish HHCS from other congenital or juvenile cataracts (Table 4) [23,24]. Elevated ferritin often precedes ocular symptoms and may be detected incidentally [5].

Several studies suggest genotype–phenotype correlations. Loop or C bulge mutations are associated with higher ferritin and denser cataracts, whereas variants outside these motifs tend to be milder [2,3,15,16]. Our findings align with other loop and C-bulge region variants, including c.-167C>T, c.-161C>T, and c.-160A>G, which disrupt IRP–IRE binding and result in moderate to high ferritin levels (typically 800–2000 µg/L) and early cataract onset [2,3,16,17]. Serum ferritin levels in our proband and affected relatives fall within this range, all exceeding 1100 µg/L (Table 1). In our family, three of four HHCS patients were diagnosed with cataracts in the neonatal period and one in early childhood, requiring surgery during adolescence (12–19 years). The youngest patient, a 6-year-old girl, is currently undergoing occlusion therapy (Table 1). Intra-familial variability reported in the literature suggests the presence of additional genetic or environmental modifiers influencing disease severity [2,3,21].

A Brazilian series described patients with c.-157G>A plus *HFE* H63D who had ferritin up to 4900 ng/mL, compared to ~1000 ng/mL in relatives with only the *FTL* mutation [21]. Co-inheritance of HHCS with *HFE* variants was also reported in a Czech family [10] and contributed to misdiagnosis as HH [12]. In our family, three of four affected members were *HFE* H63D heterozygotes, but their ferritin and hepcidin did not differ significantly. This is not unexpected, as H63D is a low-penetrance allele and usually pathogenic only in homozygotes or compound heterozygotes with C282Y [6,12]. Further studies are needed to clarify whether HFE modifies HHCS severity, particularly in adult males with HHCS, in whom *HFE*-related HH shows higher penetrance [12,13].

The main diagnostic challenge is misclassification as HH in patients with isolated hyperferritinemia [2,6]. For example, the proband’s father was considered to have HH for many years, despite a family history of cataracts, until his daughter’s evaluation prompted correct testing. This underlines how even long-standing familial cataracts may go unrecognized as HHCS without targeted genetic testing. Unlike HH, HHCS presents with normal iron indices, normal hepcidin, and no iron deposition [6]. Misdiagnosis leads to inappropriate phlebotomy or chelation, which depletes iron and causes anemia without correcting hyperferritinemia [19,25]. Severe complications, such as acute hyperammonemia, have been reported following phlebotomy in HHCS [10]. To aid in accurate differentiation, we provide a concise comparison of HHCS, adult-onset HH (related to *HFE* or *TFR2* mutations), and juvenile HH (related to *HJV* or *HAMP* mutations) (Table 5) [12,14], highlighting the clinical and biochemical distinctions critical for correct diagnosis and management.

Diagnostic work-up should include ferritin measurement in early-onset cataracts and ophthalmologic evaluation in unexplained hyperferritinemia. Normal transferrin saturation helps distinguish HHCS from secondary causes (inflammation, liver disease, infection, autoimmune, metabolic disorders, malignancy) [6]. Molecular testing of the *FTL* gene provides definitive confirmation of HHCS.

Given the autosomal dominant inheritance pattern, genetic counseling is strongly recommended. A structured family counseling plan should include an explanation of genetic risk, a detailed family history assessment, targeted genetic testing, clinical monitoring for early cataract detection, symptomatic management, reproductive counseling, and education/support for affected families. Identification of an *FTL* mutation enables predictive testing of at-risk relatives and early ophthalmologic monitoring. Counseling reassures families of the benign nature of hyperferritinemia, prevents unnecessary treatment, and provides guidance to reproductive decision-making. Greater awareness among pediatricians, ophthalmologists, and hematologists will improve case detection and outcomes.

Management of HHCS is limited to cataract surgery once visual impairment develops. Cataracts usually appear in childhood but progress slowly (Table 4), with surgery often delayed until adulthood. Importantly, hyperferritinemia requires no treatment. Clinicians’ awareness of this syndrome is crucial to prevent misdiagnosis and avoid inappropriate, potentially harmful iron-depletion therapy.

## 5. Conclusions

Our case emphasizes the diagnostic value of detailed family history and molecular testing in patients with isolated hyperferritinemia, particularly in younger individuals. This report represents the fourth Czech family described with HHCS, contributing valuable regional data.

Increased clinical awareness of HHCS is crucial. Ophthalmologists should consider ferritin testing in patients with unexplained cataracts and a suggestive family history, while hematologists should evaluate for ocular involvement in cases of persistent hyperferritinemia with normal transferrin saturation. Integration of interdisciplinary collaboration and genetic diagnostics into routine practice can facilitate earlier diagnosis, appropriate management, and improved patient outcomes.

This study highlights the translational relevance of combining molecular and clinical evaluation, demonstrating how genetic diagnostics can directly inform patient care, prevent misdiagnosis, and guide decision-making in rare hematological disorders.

## Figures and Tables

**Table 1:** Clinical characteristics and laboratory parameters of the proband and family members.

	Proband	Sister	1st Brother	Father	Mother	2nd Brother
Age [years]	13	6	18	51	46	8
Sex	F	F	M	M	F	M
*FTL* mutation	c.-168G>C/wt	c.-168G>C/wt	c.-168G>C/wt	c.-168G>C/wt	wt	wt
*HFE* mutation	H63D/wt	H63D/wt	wt	H63D/wt	wt	H63D/wt
Ferritin [ng/mL] (ref. values: 10–322 ^#^)	1418 ± 265	1333	1202	1847	-	-
sFe [uM/L] (ref. values: 10.6–28.3)	18.2	23.1	12.5	25.6	-	-
TIBC [μM/L] (ref. values: 44.8–76.1)	73.8	67.4	70.7	64.0	-	-
TSAT [%] (ref. values: 20–50)	24.7	34.3	17.7	40	.	-
Hepcidin [ng/mL] (ref. values: 5.92 ± 3.75 *)	1.98	2.61	0.45	10.60	2.01	0.87
Age of cataract dg.	Newborn	Newborn	Newborn	Early childhood	-	-
Age of cataract surgery [years]	12	-	-	19	-	-
Remark/status	-	Occlusion therapy ongoing	Awaiting surgery		-	-

Abbreviations: ref. values—reference values; sFe—serum iron; TIBC—Total Iron-Binding Capacity; TSAT—transferrin saturation; dg.—diagnosis. The mutation nomenclature follows the HGVS guidelines. ^#^ ferritin values [ng/mL]—children: 10–291, adult males: 22–322; * hepcidin values [ng/mL] from healthy controls analyzed in our laboratory—children: 1.90 ± 0.21 (*n* = 2), adolescents: 3.68 ± 0.76 (*n* = 3), adult males: 8.13 ± 3.83 (*n* = 12), adult females: 4.84 ± 3.51 (*n* = 6); hepcidin values for patients with IRIDA (Iron Refractory Iron Deficiency Anemia): 52.2 ± 9.1 ng/mL (*n* = 3).

**Table 2:** Mutations reported in HHCS.

Mutation	Alternate/Old Names	Structural/Functional Notes
Point Mutations		
c.-110C>T	+90C>U	Downstream IRE*.*
c.-144A>T	Paris + 56A>U	Lower stem; alters stem base pairing, impacts hairpin conformation and/or stability.
c.-148G>C	Heidelberg + 52G>C	
c.-149G>C	Torino + 51G>C	
c.-150C>A	+50C>A	Lower stem; affects C bulge geometry.
c.-151A>G, C	Ghent + 49A>G; + 49A>C	Upper stem; alters critical stem region for IRP binding.
c.-153G>A	Paris + 47G>A	
c.-154T>G	+46U>G	
c.-157G>A	Salt Lake City + 43G>A	Hexanucleotide loop; disrupts IRP binding.
c.-159G>C	Verona-1 + 41G>C	
c.-160A>G	Paris-1 or Montpellier-1 + 40A>G	
c.-161C>T, G, A	London-1 + 39C>U; Paris + 39C>G; Geelong + 39C>A	
c.-163A>T, G, C	Zaragoza + 37A>U; Milano + 37A>G; Pavia + 37A>C	Upper stem; alters critical stem region.
c.-164C>T, G, A	Badalona + 36C>U; Milano + 36C>G; London-2 + 36C>A	
c.-166T>C	Paris + 34U>C	Upper stem; affects hairpin stability.
c.-167C>T, A	Madrid or Philadelphia + 33C>U; Paris + 33C>A	C bulge; disrupts IRP binding.
c.-168G>T, C, A	Paris-2 or Milano-1 + 32G>U; Baltimore-1 + 32G>C; Pavia-1 + 32G>A	Lower stem; affects C bulge geometry.
c.-171C>G	Torino + 29C>G	Lower stem; alters stem base pairing; impacts hairpin conformation and/or stability.
c.-176T>C	+24U>C	
c.-178T>G	22U>G	
c.-182C>T	Paiva-2 + 18C>U	
c.-184C>T	+16C>U	
c.-186C>G	+14C>G	
c.-190C>T	+10C>U	Lower stem; affects stem stability.
c.-193C>G	+7C>G	Lower stem near loop base; potential IRP interaction effect.
c.-216C>A	*NA*	*FTL* promoter*.*
Deletions		
c.-44del	+176delT	3′ flanking region; affects stability.
c.-153_-152delinsCT *	Pori + 47 G>C and + 48 G>T *	Upper stem; partial elimination; alters stem integrity.
c.-158_-143del	+42_57del16	Hexanucleotide loop; partial elimination, disrupts IRP binding.
c.-162_-161del	+38_39delAC	
c.-161del	+39delC	
c.-164_-158del	Esplugues + 36_42del7	
c.-168_-165del	+32_35del4	C bulge; elimination; disrupts IRP binding.
c.-178_-173del	+22_27del6	Lower stem, partial elimination, impacts IRP binding.
c.-182_-174delinsAGGGGCCGG	+18_ + 26del9ins9	Complex deletion-insertion; disrupts IRE structure and IRP binding.
c.-190_-162del	Verona-2 + 10_38del29	Large deletion spanning stem; eliminates IRE.
c.-220_-196del	*NA*	Large deletion; new transcription start point.

Abbreviations: *NA*—not applicable; del—deletion; ins—insertion. * Before standardized HGVS rules were adopted, this variant was described as two adjacent substitutions (double nucleotide variant) [11]. Current HGVS nomenclature prefers to describe two adjacent base changes that together replace one sequence with another as a deletion-insertion (delins).

**Table 3:** Characteristics of previously reported Czech HHCS patients.

	Mutation	Inheritance	Ferritin Range * µg/L	sFe Range * µmol/L	TSAT Range * %	Age at dg.	Ophthalmological Findings	Clinical Notes
Family 1	c.-167C>T	de novo	750–1275 ^$^	8.7	15.7	P1: 7.5 y	insignificant visual lens opacities	dg. after chelation therapy and seizure due to hyperammonemia
Family 2	c.-168G>C	AD	1861–2328	14.6–24.3	20–28	P2: 43 y	premature cataract formation	co-inheritance with HFE, hyperferritinemia incidentally found during the management of pulmonary embolism
						P3: 4.5 y	bilateral lens opacities	hyperferritinemia noted during an investigation of petechiae formation
Family 3	c.-161C>T	AD	924–1000	13.8–20.1	31 ^#^	P4: 36 y	premature cataract formation	-
						P5: 3.5 y	-	hyperferritinemia found during management of joint pain and high temperature episodes

For additional details, see Moravikova J. et al. [10]. Abbreviations and symbols: AD—autosomal dominant; dg.—diagnosis; y—years; TSAT—transferrin saturation; sFe—serum iron; P—patient, * range indicates levels of iron parameters measured in affected individuals within each family, ^$^ range represents multiple measurements for P1, ^#^ TSAT available only for P5.

**Table 4:** Characteristics distinguishing different types of congenital cataracts.

Feature	HHCS-Associated Cataract	Congenital Cataract	Metabolic/Secondary Cataract
Onset	Early (childhood to adolescence)	Birth or early infancy	Variable; often pediatric or young adult
Family history	Often familial	Often familial	Varies (depends on systemic disease)
Laterality	Bilateral and symmetric	Often bilateral	Bilateral or unilateral
Cataract morphology	Crystalline opacities in the lens cortex; frosted glass appearance	Varied: nuclear, lamellar, posterior	e.g., Snowflake (diabetes), sunflower (Wilson’s)
Progression	Slowly progressive	May remain stable or progress	Variable; depends on cause
Systemic associations	↑ serum ferritin without iron overload	Genetic syndromes (e.g., Wolfram, Senger’s, Nance-Horan, or Down syndrome)	Diabetes, Wilson’s disease, myotonic dystrophy
Iron parameters	↑ ferritin, other parameters normal	Normal	Normal or variable (e.g., high in inflammation)
Inheritance	AD; *FTL* gene	AD, AR, or X-linked: lens development genes; trisomy 21	Depends on disease
Phlebotomy/ Chelation therapy	No benefit, potentially harmful; iron overload not present	Not applicable	Depends on condition (e.g., copper chelation in Wilson’s disease)
Other ocular findings	Non specific	May have microphthalmia, microcornea, or other signs	Depends on condition

For more details, see Shiels A and Hejtmancik JF [23] and Ang MJ and Afshari NA [24]. Abbreviations and symbols: ↑ = elevated; AD—autosomal dominant; AR—autosomal recessive; e.g.—for example; TSAT—transferrin saturation.

**Table 5:** Comparison of HHCS with adult-onset and juvenile HH.

Feature	HHCS	Adult HH	Juvenile HH
Gene	*FTL* (5′UTR)	*HFE*, *TFR2*	*HJV*, *HAMP*
Inheritance	AD	AR	AR
Typical onset	Childhood/adolescence	30–50 y	Childhood/adolescence
Serum ferritin	↑	↑	↑↑
Serum iron/TSAT	Normal	↑	↑↑
Hepcidin	Normal	Low-normal	Very low
Iron overload	No	Yes	Severe
Lens changes	Bilateral cataracts (early-onset)	Rare	Possible (secondary)
Main complications	Visual impairment	Organ iron deposition, cirrhosis, diabetes, arthropathy	Organ iron deposition, cirrhosis, cardiomyopathy, diabetes, hypogonadism
Management	Ophthalmologic monitoring, cataract surgery; Avoid phlebotomy/chelation	Phlebotomy	Phlebotomy ± chelation

Adult HH caused by *HFE* or *TFR2* (transferrin receptor 2) mutations and juvenile HH caused by *HJV* (hemojuvelin) or *HAMP* (hepcidin) mutations result in decreased hepcidin production, leading to increased intestinal iron absorption and recycling and systemic iron overload [14]. HFE and TFR2 act as part of the hepatocyte iron-sensing complex, while HJV directly regulates HAMP expression [14]. In contrast, HHCS caused by *FTL* mutations affects ferritin translation without altering hepcidin or systemic iron homeostasis. Abbreviations and symbols: AD—autosomal dominant; AR—autosomal recessive; y—years; TSAT—transferrin saturation; ↑ = elevated; ↑↑ = markedly elevated.

## Data Availability

Due to participant privacy considerations, the data supporting this study is not publicly available. De-identified data may be obtained from the corresponding author upon reasonable request.

## Abbreviations

The following abbreviations are used in this manuscript:

5′ UTR	5′ untranslated region
*FTL*	*ferritin light chain* gene
*HAMP*	*hepcidin antimicrobial peptide*
*HFE*	*homeostatic iron regulator*
HH	hereditary hemochromatosis
HHCS	hereditary hyperferritinemia-cataract syndrome
*HJV*	*hemojuvelin*
IRE	iron-responsive element
IRPs	iron regulatory proteins
MRI	magnetic resonance imaging
*TFR2*	*transferrin receptor 2*
